# Changes in blood catecholamines during induction of general anesthesia in patients with post-induction hypotension undergoing laparoscopic cholecystectomy: A single-center prospective cohort study

**DOI:** 10.1371/journal.pone.0305980

**Published:** 2024-06-25

**Authors:** Yi Yao, Xia Kong, Xuhui Chen, Yingying Zhang, Xueru Liu, Xiaobin Wang

**Affiliations:** 1 Department of Anesthesiology, The Affiliated Hospital of Southwest Medical University, Luzhou, Sichuan Province, China; 2 Department of Anesthesiology, Anesthesiology and Critical Care Medicine Key Laboratory of Luzhou, Southwest Medical University, Luzhou, Sichuan Province, China; Affiliated Hospital of Nantong University, CHINA

## Abstract

**Background:**

Post-induction hypotension (PIH) often occurs during general anesthesia induction. This study aimed to investigate blood catecholamine levels during induction of general anesthesia in patients with PIH undergoing laparoscopic cholecystectomy.

**Methods:**

This prospective study included 557 adult patients who underwent laparoscopic cholecystectomy under general anesthesia. PIH was defined as a greater than 20% decrease in systolic blood pressure from the pre-induction value, a systolic arterial pressure of less than 90 mmHg, or both. Plasma concentrations of epinephrine and norepinephrine during the induction of general anesthesia were determined using enzyme-linked immunosorbent assay. Multivariate logistic regression analysis evaluated the association between the clinical factors and PIH.

**Results:**

Of the 557 patients, 390 had PIH, and the remaining 167 were allocated to the non-PIH group. Changes in blood adrenaline, noradrenaline levels, or both were more pronounced in the PIH than in the non-PIH group (p<0.05). Age, body mass index, a history of hypertension, preoperative systolic blood pressure, and propofol or sufentanil dose were independent predictors of PIH.

**Conclusion:**

The changes of blood catecholamines in patients with more stable hemodynamics during the induction of general anesthesia are smaller than that in patients with post-induction hypotension.

**Trial registration:**

ChiCTR2200055549, 12/01/2022.

## Background

Laparoscopic cholecystectomy is a common procedure, with post-induction hypotension (PIH) occurring in approximately 50% of patients [1]. PIH is a common complication of anesthesia that is associated with postoperative acute renal injury [2–4], myocardial injury [5], ischemic stroke, and even death [6–9].

PIH is defined as hypotension occurring within 20 min of induction of general anesthesia [6, 10, 11]. The mechanism of PIH is likely multifactorial and remains unclear. Specific factors have been associated with PIH, including aging, emergency surgery, baseline hypovolaemia, and the use of propofol induction [12–15]. Furthermore, previous studies have reported an association between catecholamine use and PIH incidence [16]. However, changes in catecholamine levels during PIH remain unclear. Accordingly, this study aimed to analyze the changes in blood catecholamine levels related to PIH to elucidate the mechanisms of action, aiding anesthesiologists in implementing preemptive appropriate measures based on the changes in catecholamine levels in clinical practice to reduce the occurrence of PIH and improve the prognosis of patients.

## Materials and methods

The Ethics Committee of the Affiliated Hospital of Southwest Medical University approved the methods and procedures for this prospective cohort study (approval number: KY2021293, S1 and S3 Files). The trial was registered at the Chinese Clinical Trial Registration Center (ChiCTR2200055549, 12/01/2022) and was conducted between February 28, 2022, and November 31, 2022. Participants provided consent for the use of their anonymized medical data for research (S2 File), this study was conducted according to the principles expressed in the Declaration of Helsinki.

### Study design

Eligible patients who underwent elective laparoscopic cholecystectomy under general anesthesia were ≥18 years of age and had an American Society of Anesthesiologists (ASA) score of I–III. Exclusion criteria were as follows: conversion to open surgery; severe preoperative comorbidities, including a New York Heart Association class ≥IV, moderate-to-severe impairment of pulmonary ventilation, Child C grade of liver function, and stage III renal insufficiency; history of peripheral arterial disease or atherosclerosis; opioid abuse or addiction to alcohol and other drugs; cardiac rhythm other than sinus and cardiomyopathy; as well as poor compliance and loss to follow-up (S4–S6 Files).

### Anesthesia and surgical protocols

All patients underwent standard preoperative fasting and were routinely monitored in the operating room. Intravenous anesthesia, comprising sufentanil (0.2–0.4 μg kg^−1^), propofol (1.5–2.5 mg kg^−1^), and CIS atracurium (0.1–0.3 mg kg^−1^) sequentially, was administrated. Subsequently, endotracheal intubation and mechanical ventilation were performed, and inhalation anesthesia with sevoflurane was maintained.

During induction, PIH was defined as a greater than 20% decrease in systolic blood pressure (SBP) from the pre-induction value, a systolic arterial pressure of less than 90 mmHg, or both. Appropriate treatment was provided by the anesthesiologist, which included fluid rehydration and intravenous injection of metaraminol bitartrate (0.5 mg) or methoxyamine (1 mg). Hypertension, defined as SBP >160 mmHg, was properly managed by the anesthesiologist.

**Fig 1:**
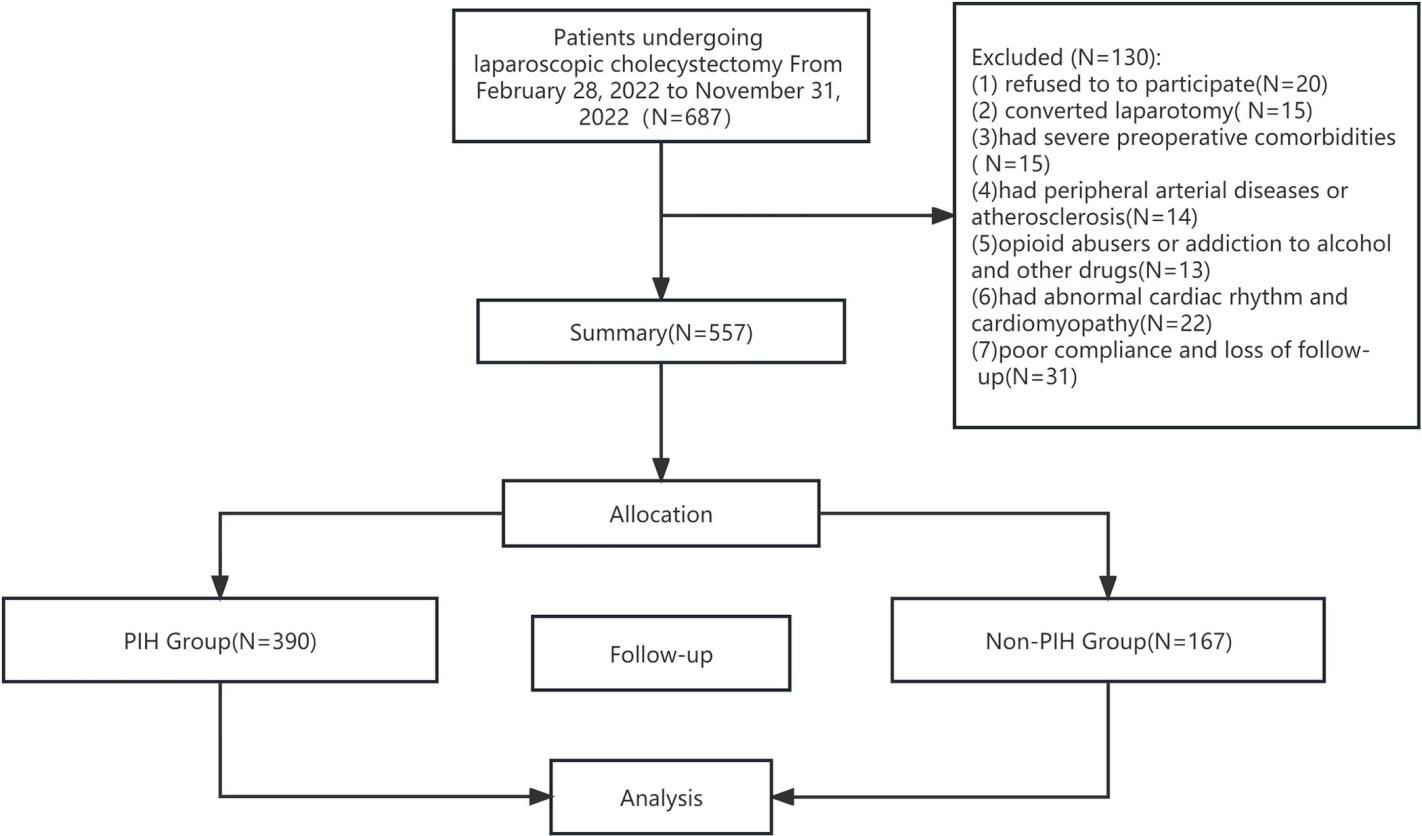
Flow chart of patient inclusion PIH: Post-induction hypotension.

Indications for placing and removing drainage tubes were based on previous studies [17]. If a patient had an abdominal drainage tube, it was removed 24–48 h after surgery in the absence of bile or bleeding. The main technical route used for surgery is shown in Fig 1.

### Main indicators

The main indicators for evaluation were the incidence of PIH and blood catecholamine (adrenaline and norepinephrine) levels during anesthesia induction. The criterion for PIH was at least one episode of a greater than 20% decrease in SBP from the pre-induction value or systolic arterial pressure of less than 90 mmHg within 20 min after induction. The occurrence rate over the period of observation was calculated. To ensure blood perfusion in important organs, measures such as volume supplementation and vasopressor drug use were implemented immediately after PIH onset. Therefore, the duration of PIH was not included in the observation period. Radial artery blood (>5 mL) was collected from all patients at three time points: before anesthesia induction (T_0_), immediately after anesthesia induction (T_1_), and 3–5 min after tracheal intubation (T_2_). Heart rate (HR), SBP, and diastolic blood pressure (DBP) were recorded. Blood was collected and stored as previously described [18]. Plasma concentrations of epinephrine and norepinephrine were determined using enzyme-linked immunosorbent assay.

Based on the results of this experiment, we set a ratio for the hypotension and non-hypotension groups and the sample size using the software PASS 2011. The corresponding quantity was randomly selected from all samples to detect adrenaline and norepinephrine concentrations using radioimmunoassay.

### Secondary indicators

The secondary indicators encompassed risk factors for PIH, which included pre-anesthesia data (age, sex, body mass index [BMI], ASA grade, chronic diseases, history of hypertension, creatinine level, eGFR [estimated Glomerular Filtration Rate], and baseline blood pressure) and induction period data (dose of propofol, sufentanil, or CIS atracurium and initial carbon dioxide pressure (P_ET_CO_2_)]. The median dose of the induction drug was calculated, and the number of patients receiving a dose greater or less than the median dose was counted. The initial P_ET_CO_2_ value was calculated based on the electronic anesthesia system records. Additionally, postoperative data, such as sleep quality, sedation classification, time to first exhaustion or defecation, nausea and vomiting, shoulder and back pain, headache, urinary retention, and time to discharge, were recorded. The Ramsay score was used to grade sedation. Sleep status was evaluated using the Athens Insomnia Scale (AIS), with a score ≥4 indicating insomnia. Data were collected by two researchers through a review of electronic medical charts and interviews with patients, family members, and surgeons. Both researchers completed standardized training during the interview process. The data were audited regularly to ensure reliability and quality.

### Statistical analysis

Descriptive statistics were used to summarize the variables, with counts and percentages (%) used for categorical data and mean ± standard deviation or median and quartile range for continuous data, as appropriate for data distribution. Patients were classified into PIH and non-PIH groups. Between-group differences in continuous variables were evaluated using an independent t-test for normally distributed data and the Mann–Whitney U-test for variables with a non-normal distribution. The chi-squared test was used to compare categorical variables. Variables with a between-group difference of p≤0.1 were included in a multivariate logistic regression model to identify independent risk factors for PIH. Adrenaline and noradrenaline levels were compared using two-way repeated-measures analysis of variance, considering the interaction between time and group. All tests were two-sided, and the significance level was set at p<0.05. All statistical analyses were performed using R 4.0.5.

## Results

### Characteristics of the study sample and perioperative data

Of the 687 patients eligible for the study, 91 and 39 patients in the PIH and non-PIH groups, respectively, were excluded for the following reasons: conversion to laparotomy, missing anesthesia records, and loss to follow-up. Accordingly, 557 patients were included in the final analysis(S1 Table): 390 and 167 in the PIH and non-PIH groups, respectively (70% vs. 30%). The perioperative data for the PIH and non-PIH groups are shown in Table 1. Significant between-group differences were identified for the following factors: age, BMI, creatinine level, history of hypertension, preoperative SBP, DBP, HR, post-induction SBP, and dosage of propofol or sufentanil. Additionally, between-group differences were noted in the following postoperative factors: time of first defecation, headache, and urinary retention.

**Table 1:** Perioperative data of patients.

Variables	Total (n = 557)	Non-PIH (n = 167)	PIH (n = 390)	*P-*value
**Baseline variables**				
Gender, n (%)				0.08
male	194 (34.8)	49 (29.3)	145 (37.2)	
female	363 (65.2)	118 (70.7)	245 (62.8)	
Age (year)	50.9±13.1	44.2±12.9	53.8±12.1	<0.001
BMI (kg/m^2^)	23.5±3.1	24.5±3.3	23.0±3.0	<0.001
ASA, n (%)				0.62
I	519 (93.2)	156 (93.4)	363 (93.1)	
II	23 (4.1)	8 (4.8)	15 (3.8)	
III	15 (2.7)	3 (1.8)	12 (3.1)	
Chronic diseases, n (%)				0.15
No	318 (57.1)	103 (61.7)	215 (55.1)	
Yes	239 (42.9)	64 (38.3)	175 (44.9)	
The history of hypertension, n (%)				<0.001
No	421 (75.6)	154 (92.2)	267 (68.5)	
Yes	136 (24.4)	13 (7.8)	123 (31.5)	
Creatinine(μmoI/L)	62.0±17.2	59.7±19.7	63.0±16.0	0.04
eGFR <90mL/min				0.09
No	479 (86.0)	150 (89.8)	329 (84.4)	
Yes	78 (14.0)	17 (10.2)	61 (15.6)	
Initial value of PETCO2 (mmHg)	33.5±5.1	33.6±8.1	32.5±3.8	0.60
**Intraoperative condition**				
Preoperation (T0)				
SBP (mmHg)	134.1±20.9	123.7±18.1	138.6±20.4	<0.001
DBP (mmHg)	78.3±11.3	74.0±11.1	80.1±10.8	<0.001
HR (times/min)	76.2±11.3	74.6±11.9	76.9±10.9	0.02
Post-induction(T1)				
SBP (mmHg)	107.5±16.1	111.3±15.5	105.9±16.1	<0.001
DBP (mmHg)	64.0±10.9	65.1±10.6	63.6±11.0	0.15
HR (times/min)	66.1±10.5	65.8±9.859	66.3±10.7	0.63
Post-intubation(T2)				
SBP (mmHg)	109.3±16.6	113.5±17.7	107.5±15.8	<0.001
DBP (mmHg)	64.5±11.1	65.7±11.3	64.0±11.0	0.09
HR (times/min)	63.7±9.9	64.6±9.5	63.4±10.1	0.19
Propofol (doses in mg/kg)				<0.001
Mean dose	1.7±0.4	1.6±0.4	1.8±0.4	
Doses >2 mg/kg, n (%)	176 (31.6)	31 (18.6)	145 (37.2)	
Sufentanil (doses in μg/kg)				<0.001
Mean dose	19.7±4.9	18.5±4.6	20.1±4.9	
Doses >0.3 ug/kg, n (%)	352 (63.2)	82 (49.1)	270 (69.2)	
Cis-atracurium (doses in mg/kg)				0.49
Mean dose	11.10±2.3	11.0±2.5	11.1±2.2	
Doses >0.2 mg/kg, n (%)	352 (63.2)	82 (49.1)	270 (69.2)	
**Postoperative outcomes**				
Ramsay score after surgery (points)	1.0 [1.0, 3.0]	1.0 [1.0, 2.0]	1.0 [1.0, 3.0]	0.10
Time of first exhaust, n (%)				0.09
The day of surgery	13 (2.3)	7 (4.2)	6 (1.5)	
The first day after surgery	467 (83.8)	132 (79.0)	335 (85.9)	
The second day after surgery	69 (12.4)	24 (14.4)	45 (11.5)	
The third day after surgery	8 (1.4)	4 (2.4)	4 (1.0)	
Time of first defecation, n (%)				0.01
The first day after surgery	6 (1.1)	2 (1.2)	4 (1.0)	
The second day after surgery	196 (35.2)	76 (45.6)	120 (30.8)	
The third day after surgery	355 (63.7)	89 (53.2)	266 (68.2)	
Time of first eating, n (%)				0.65
The day of surgery	7 (1.3)	3 (1.8)	4 (1.0)	
The first day after surgery	511 (91.7)	155 (92.8)	356 (91.3)	
The second day after surgery	36 (6.5)	8 (4.8)	28 (7.2)	
The third day after surgery	3 (0.5)	1 (0.6)	2 (0.5)	
Headache, n (%)				<0.001
No	305 (54.8)	143 (85.6)	162 (41.5)	
Yes	252 (45.2)	24 (14.4)	228 (58.5)	
Nausea and vomiting, n (%)				0.68
No	456 (81.9)	135 (80.8)	321 (82.3)	
Yes	101 (18.1)	32 (19.2)	69 (17.7)	
Shoulder and back pain, n (%)				0.23
No	419 (75.2)	120 (71.9)	299 (76.7)	
Yes	138 (24.8)	47 (28.1)	91 (23.3)	
Urinary retention, n (%)				<0.001
No	340 (61.0)	147 (88.0)	193 (49.5)	
Yes	217 (39.0)	20 (12.0)	197 (50.5)	
Postoperative insomnia, n (%)				0.93
No	132 (23.7)	40 (24.0)	92 (23.6)	
Yes	425 (76.3)	127 (76.0)	298 (76.4)	
Time of abdominal drainage tube extraction, n (%)				0.97
No drainage tube	403 (72.3)	121 (72.5)	282 (72.3)	
The first day after surgery	105 (18.9)	32 (19.2)	73 (18.7)	
The second day after surgery	49 (8.8)	14 (8.3)	35 (9.0)	
Time of discharge, n (%)				0.31
The day of surgery	188 (33.8)	64 (38.3)	124 (31.8)	
The first day after surgery	360 (64.6)	100 (59.9)	260 (66.7)	
The second day after surgery	9 (1.6)	3 (1.8)	6 (1.5)	

As shown in Table 2, the following factors were independently associated with PIH: age [odds ratio (OR), 1.04; 95% confidence interval (CI), 1.01–1.05; p<0.001]; BMI (OR, 0.87; 95%CI, 0.80–0.93; p<0.001); history of hypertension(OR, 3.07; 95%CI, 1.60–6.24; p = 0.001); preoperative SBP (OR, 1.04; 95% CI, 1.02–1.05; p<0.001); propofol dose >2 mg/kg (OR, 2.47; 95%CI, 1.47–4.24; p *=* 0.001); and sufentanil dose > 0.3 ug/kg (OR, 2.37; 95%CI; 1.53–3.68; p*<*0.001).

**Table 2:** Analysis of risk factors of PIH.

	Univariate model		Multivariate model	
**Variable**	**OR_95CI**	***P-*value**	**OR_95CI**	***P-*value and Estimate**
Gender	0.70 [0.47, 1.03]	0.08		
Age	1.06 [1.04, 1.08]	<0.001	1.04 [1.01–1.05]	<0.001 and 0.037
BMI	0.86 [0.81, 0.91]	<0.001	0.87 [0.80–0.93]	<0.001 and -0.145
ASA	1.13 [0.68, 1.86]	0.64		
Chronic diseases	1.31 [0.90, 1.89]	0.15		
Preoperative hypertension	5.46 [2.98, 9.99]	<0.001	3.07 [1.60–6.24]	0.001 and 1.121
Creatinine	1.01 [1.00, 1.02]	0.04		
eGFR	1.64 [0.92, 2.89]	0.09		
Preoperative SBP	1.04 [1.03, 1.05]	<0.001	1.04 [1.02–1.05]	<0.001 and 0.037
Preoperative DBP	1.05 [1.03, 1.07]	<0.001		
Preoperative HR	1.02 [1.00, 1.03]	0.02		
Initial value of P_ET_CO_2_	0.98 [0.92, 1.04]	0.59		
Propofol > 2 mg/kg	2.60 [1.67, 4.03]	<0.001	2.47 [1.47–4.24]	0.001 and 0.905
Sufentanil > 0.3 ug/kg	2.33 [1.60, 3.38]	<0.001	2.37 [1.53–3.68]	<0.001and 0.862
Cis-atracurium> 0.2 mg/kg	0.86 [0.56,1.30]	0.49		

### Catecholamine levels and PIH

Based on the results of this study, the incidence of hypotension was 70%. We set a ratio of 7:3 for the hypotension and non-hypotension groups, assuming that α = 0.05, β = 0.20, requiring at least 50 plasma samples from patients with PIH and 22 plasma samples from patients without PIH. Assuming a loss-to-follow-up rate of 10%, 80 study participants were required. Therefore, based on double-blind random sampling, plasma samples from 56 and 24 patients in the induced hypotension and non-induced hypotension groups, respectively, were selected for the detection of adrenaline and norepinephrine concentrations using radioimmunoassays(S2 Table).

As shown in Table 3, the two-way repeated-measures analysis of variance between the PIH and non-PIH groups showed that there were statistical differences at different time points; the level of adrenaline in the PIH group was significantly higher than that in the non-PIH group at T_2_ (p<0.05), and norepinephrine levels in the PIH group were significantly higher than those in the non-PIH group at T_1_ and T_2_ (p<0.05). Further comparison of the differences in values showed that the increase in norepinephrine at T_1_ and T_2_ and the increase in epinephrine at T_2_ were greater in PIH group. Compared with T_0_ before anesthesia induction, the levels of epinephrine and norepinephrine were significantly increased in both groups at T_2_ (p<0.05), and the levels of norepinephrine were significantly increased in both groups at T_1_ (p<0.05).

**Table 3:** Comparison of adrenaline (pg/mL) and noradrenaline (ng/mL) levels.

	Non-PIH (*n* = 24)	PIH (*n* = 56
EPI		
T_0_	1088.9 [931.3, 1232.4]	1056.0 [793.5, 1342.9]
T_1_	1030.9 [830.2, 1164.5]	1092.4 [876.9, 1661.2]
T_2_	1282.6 [1124.1, 1499.7]^#^	1597.8 [1271.7, 2162.0] * ^#^
T_1_-T_0_	−86.2 [-264.1, 94.2]	70.8 [−142.9, 396.6]
T_2_-T_0_	135.2 [66.8, 267.3]	446.4 [199.8, 950.3]*
NE		
T_0_	3.9 [3.3, 6.2]	6.5 [3.2, 9.7]
T_1_	5.3 [4.1, 7.0]^#^	8.2 [5.3, 15.9] * ^#^
T_2_	6.7 [4.5, 8.2]^#^	9.0 [5.1, 27.9] * ^#^
T_1_-T_0_	0.8 [0.2, 1.8]	2.2 [0.5, 4.5]*
T_2_-T_0_	1.6 [0.5, 3.5]	2.7 [1.5, 8.9]*

EPI, adrenaline; NE, noradrenaline; T_0_, before anesthesia induction; T_1_, immediately after anesthesia induction; T_2_:3–5 minutes after tracheal intubation. Vs Non-PIH

*p<0.05; Vs T_0_

^#^ p<0.05.

## Discussion

This study observed more pronounced changes in adrenaline, noradrenaline levels, or both in the PIH group than in the non-PIH group during induction of general anesthesia. One possible explanation is that the lower blood pressure in the PIH group may have activated the renin-angiotensin and vasopressin systems, increasing the secretion of norepinephrine and epinephrine in the PIH group [19]. Injurious stimuli such as tracheal intubation and surgical incision trigger stress reactions mediated via the sympathetic-adrenal medullary axis, releasing catecholamines. Accordingly, blood levels of catecholamine hormones are frequently used as reliable indicators of stress during surgery [20]. Our finding of higher plasma norepinephrine and epinephrine levels at T_2_ in the PIH and non-PIH groups compared to the baseline value (T_0_) may reflect the noxious stimulation generated during tracheal intubation. Plasma norepinephrine levels in both groups at T_1_ were higher than those at baseline (T_0_), potentially attributed to the notable decrease in the patient’s blood pressure during anesthesia induction, activating the stress system and increasing the compensatory effect of norepinephrine [21].

This study identified age, BMI, a history of hypertension, preoperative SBP, and propofol and sufentanil doses as independent predictors of PIH in patients who underwent laparoscopic cholecystectomy under general anesthesia. Older patients had a higher risk for PIH [22–24]. The negative correlation between BMI and PIH likely reflects a reduction in autonomic nervous activity and retention of salt and water in patients with a low BMI [25, 26]. Hypertension as a risk factor for PIH has been previously reported [27]. To explore the influence of the induction drug dose on the risk of PIH, we compared the incidence of PIH among subgroups of patients who received doses greater or less than the median dose. The median dosages are as follows: propofol, 2 mg/kg; sufentanil, 0.3 μg/kg; and CIS atracurium, 0.2 mg/kg [13]. Higher doses of propofol or sufentanil are risk factors for PIH, consistent with the dose-dependent relationship previously described for these drugs and intraoperative hypotension [10]. The decrease in blood pressure caused by the above factors primarily stems from reduced sympathetic activity [28, 29], directly affecting the vascular smooth muscle, which leads to arterial vessel dilation and venous relaxation [30–32]. The study revealed a positive association between higher preoperative SBP and PIH. Elevated preoperative SBP could be linked to the “white coat” effect and increased sympathetic excitability before surgery [33]; however, the specific reasons need further exploration.

Additionally, prolonged exhaustion, defecation, and eating were mainly related to older age, drainage tube placement, and refusal to eat owing to severe digestive tract reactions. Notably, these postoperative factors extend the length of the hospital stay. Moreover, a higher incidence of postoperative headache was observed in the PIH group than in the non-PIH group, which may be related to the negative effect of hypotension on cerebral blood flow, contributing to the potential mechanism of PIH-related headache [34].

The limitations of this study should be acknowledged. First, these independent risk factors for PIH need further confirmation in multicenter studies with larger sample sizes. Second, because of the short hospital stay of the patients who underwent laparoscopic cholecystectomy, follow-up was difficult, resulting in insufficient long-term follow-up results.

In summary, during the induction of general anesthesia, more obvious changes in adrenaline, noradrenaline levels, or both were observed in the PIH group than in the non-PIH group. Age, BMI, history of hypertension, preoperative SBP, and propofol or sufentanil dose were independent predictors of PIH in patients who underwent laparoscopic cholecystectomy under general anesthesia. Our findings highlights the importance of preoperative risk stratification and tailored management approaches for at-risk patients.

## Supporting information

S1 File The approval of clinical trial ethics committee. (PDF)

S2 File Translation-informed consent form. (PDF)

S3 File Translation-the approval of clinical trial ethics committee. (PDF)

S4 File Research plan for induced hypotension project. (PDF)

S5 File Human participants research checklist. (PDF)

S6 File CONSORT checklist. (PDF)

S7 File Certificate of editing. (PDF)

S1 Table Raw data 1. (XLSX)

S2 Table Raw data 2. (XLSX)

## Abbreviations

PIH: Post-induction hypotension
BMI: Body mass index
Pre: Preoperation
ASA: American Society of Anesthesiologists
MAP: Mean artery pressure
HR: Heart rate
DBP: Diastolic blood pressure
SBP: Systolic blood pressure
P_ET_CO_2_: End-tidal carbon dioxide

## References

[1] Lee J, Woo J, Kang AR, Jeong Y-S, Jung W, Lee M, et al. Comparative analysis on machine learning and deep learning to predict post-induction hypotension. Sensors. 2020;20:4575. doi: 10.3390/s20164575 32824073 PMC7472016

[2] Maheshwari K, Turan A, Mao G, Yang D, Niazi AK, Agarwal D, et al. The association of hypotension during non‐cardiac surgery, before and after skin incision, with postoperative acute kidney injury: a retrospective cohort analysis. Anaesthesia. 2018;73:1223–8. doi: 10.1111/anae.14416 30144029

[3] Vaara ST, Bellomo R. Postoperative renal dysfunction after noncardiac surgery: Current Opinion in Critical Care. 2017;23:440–6. 10.1097/MCC.0000000000000439 28820797

[4] Mathis MR, Naik BI, Freundlich RE, Shanks AM, Heung M, Kim M, et al. Preoperative risk and the association between hypotension and postoperative acute kidney injury. Anesthesiology. 2020;132:461–75. doi: 10.1097/ALN.0000000000003063 31794513 PMC7015776

[5] Salmasi V, Mascha EJ. Relationship between Intraoperative Hypotension, Defined by either reduction from baseline or absolute thresholds, and acute kidney and myocardial injury after noncardiac surgery: a retrospective cohort analysis. PERIOPERATIVE MEDICINE. 2017;126:47–65. doi: 10.1097/ALN.0000000000001432 27792044

[6] Südfeld S, Brechnitz S, Wagner JY, Reese PC, Pinnschmidt HO, Reuter DA, et al. Post-induction hypotension and early intraoperative hypotension associated with general anaesthesia. British Journal of Anaesthesia. 2017;119:57–64. doi: 10.1093/bja/aex127 28974066

[7] Sessler DI, Bloomstone JA, Aronson S, Berry C, Gan TJ, Kellum JA, et al. Perioperative quality initiative consensus statement on intraoperative blood pressure, risk and outcomes for elective surgery. British Journal of Anaesthesia. 2019;122:563–74. doi: 10.1016/j.bja.2019.01.013 30916004

[8] Althoff FC, Agnihotri A, Grabitz SD, Santer P, Nabel S, Tran T, et al. Outcomes after endoscopic retrograde cholangiopancreatography with general anesthesia versus sedation. British Journal of Anaesthesia. 2021;126:191–200. 33046219 10.1016/j.bja.2020.08.057

[9] Smischney NJ, Shaw AD, Stapelfeldt WH, Boero IJ, Chen Q, Stevens M, et al. Postoperative hypotension in patients discharged to the intensive care unit after non-cardiac surgery is associated with adverse clinical outcomes. Crit Care. 2020;24:682. doi: 10.1186/s13054-020-03412-5 33287872 PMC7720547

[10] Tarao K, Daimon M, Son K, Nakanishi K, Nakao T, Suwazono Y, et al. Risk factors including preoperative echocardiographic parameters for post-induction hypotension in general anesthesia. Journal of Cardiology. 2021;78:230–6. doi: 10.1016/j.jjcc.2021.03.010 33838982

[11] Wani TM, Hakim M, Ramesh A, Rehman S, Majid Y, Miller R, et al. Risk factors for post-induction hypotension in children presenting for surgery. Pediatr Surg Int. 2018;34:1333–8. doi: 10.1007/s00383-018-4359-5 30350110

[12] Engel D, Beilstein CM, Löffel LM, Wuethrich PY. The impact of fluid optimisation before induction of anesthesia on hypotension after induction. Anaesthesia. 2020;75:1402–1402. 32578194 10.1111/anae.15080

[13] Reich DL, Hossain S, Krol M, Baez B, Patel P, Bernstein A, et al. Predictors of hypotension after induction of general anesthesia: anesthesia & analgesia. 2005;101:622–8. 10.1213/01.ANE.0000175214.38450.91 16115962

[14] Okamura K. Pre-anesthetic ultrasonographic assessment of the internal jugular vein for prediction of hypotension during the induction of general anesthesia. Journal of Anesthesia. 2019;33:612–9. doi: 10.1007/s00540-019-02675-9 31451896

[15] Lankadeva YR, May CN, Bellomo R, Evans RG. Role of perioperative hypotension in postoperative acute kidney injury: a narrative review. British Journal of Anaesthesia. 2022;128:931–48. doi: 10.1016/j.bja.2022.03.002 35465952

[16] Macnab MSP, Manninen PH, Lam AM, Gelb AW. The stress response to induced hypotension for cerebral aneurysm surgery: a comparison of two hypotensive techniques. Can J Anaesth. 1988;35:111–5. doi: 10.1007/BF03010648 3281763

[17] Picchio M, De Cesare A, Di Filippo A, Spaziani M, Spaziani E. Prophylactic drainage after laparoscopic cholecystectomy for acute cholecystitis: a systematic review and meta-analysis. Updates Surg. 2019;71:247–54. doi: 10.1007/s13304-019-00648-x 30945148

[18] Tu W, Yuan H, Zhang S, Lu F, Yin L, Chen C, et al. Influence of anesthetic induction of propofol combined with esketamine on perioperative stress and inflammatory responses and postoperative cognition of elderly surgical patients. Am J Transl Res. 2021;13:1701–9. 33841692 PMC8014402

[19] Schulte E, Ziegler D, Philippi-Höhne C, Kaczmarczyk G, Boemke W. Angiotensin-converting enzyme inhibition and blood pressure response during total intravenous anesthesia for minor surgery. Acta Anaesthesiologica Scandinavica. 2011;55:435–43. 21391923 10.1111/j.1399-6576.2011.02409.x

[20] Fukusaki M, Maekawa T, Kobayashi I, Hara T, Sumikawa K. Catecholamine and renin-angiotensin response during controlled hypotension induced by prostaglandin E1 combined with hemodilution during isoflurane anesthesia. Journal of Clinical Anesthesia. 1997;9:321–7. doi: 10.1016/s0952-8180(97)00011-1 9195357

[21] Helander EM, Webb MP, Menard B, Prabhakar A, Helmstetter J, Cornett EM, et al. Metabolic and the surgical stress response considerations to improve postoperative recovery. Curr Pain Headache Rep. 2019;23:33. doi: 10.1007/s11916-019-0770-4 30976992

[22] Zivaljevic V. Risk factors for intraoperative hypotension during thyroid surgery. Med Sci Monit. 2013;19:236–41. doi: 10.12659/MSM.883869 23548975 PMC3659157

[23] Nakasuji M, Nakasuji K. Causes of arterial hypotension during anesthetic induction with propofol investigated with perfusion index and clearsight in young and elderly patients. Minerva Anestesiol. 2021;87:640–7. 33688696 10.23736/S0375-9393.21.15226-5

[24] Chen B. A systematic review of risk factors for postinduction hypotension in surgical patients undergoing general anesthesia. R An. 2021;25:7044–50. doi: 10.26355/eurrev_202111_27255 34859868

[25] Coimbra S, Brandão Proença J, Santos-Silva A, Neuparth MJ. Adiponectin, leptin, and chemerin in elderly patients with type 2 diabetes mellitus: a close linkage with obesity and length of the disease. BioMed Research International. 2014;2014:1–8. doi: 10.1155/2014/701915 25105135 PMC4101968

[26] Nakamura T, Suzuki M, Ueda M, Hirayama M, Katsuno M. Lower body mass index is associated with orthostatic hypotension in Parkinson’s disease. Journal of the Neurological Sciences. 2017;372:14–8. doi: 10.1016/j.jns.2016.11.027 28017201

[27] Hojo T, Kimura Y, Shibuya M, Fujisawa T. Predictors of hypotension during anesthesia induction in patients with hypertension on medication: a retrospective observational study. BMC Anesthesiol. 2022;22:343. doi: 10.1186/s12871-022-01899-9 36368916 PMC9650866

[28] Alizadeh R, Fard ZA. Renal effects of general anesthesia from old to recent studies. Journal Cellular Physiology. 2019;234:16944–52. doi: 10.1002/jcp.28407 30843210

[29] Araújo AM, Machado HS, Falcão AC, Soares-da-Silva P. Reliability of body-weight scalars on the assessment of propofol induction dose in obese patients. Acta Anaesthesiol Scand. 2018;62:464–73. doi: 10.1111/aas.13039 29159892

[30] Jang CH, Cho YB, Lee JS, Kim GH, Jung W-K, Pak SC. The effect of propofol infusion with topical epinephrine on cochlear blood flow and hearing: an experimental study. International Journal of Pediatric Otorhinolaryngology. 2016;91:23–6. doi: 10.1016/j.ijporl.2016.10.006 27863636

[31] Darvall J, Vijayakumar R, Leslie K. Prewarming neurosurgical patients to minimize hypotension on induction of anesthesia: a randomized trial. Can J Anesth/J Can Anesth. 2016;63:577–83. doi: 10.1007/s12630-016-0601-6 26858092

[32] Saugel B, Bebert E-J, Briesenick L, Hoppe P, Greiwe G, Yang D, et al. Mechanisms contributing to hypotension after anesthetic induction with sufentanil, propofol, and rocuronium: a prospective observational study. J Clin Monit Comput. 2022;36:341–7. doi: 10.1007/s10877-021-00653-9 33523352 PMC9122881

[33] Koutsaki M, Thomopoulos C, Achimastos A, Kallistratos M, Batistaki C, Chatziagelaki E, et al. Perioperative SBP changes during orthopedic surgery in the elderly: clinical implications. Journal of Hypertension. 2019;37:1705–13. doi: 10.1097/HJH.0000000000002085 30950973

[34] Matsota PK, Christodoulopoulou TC, Batistaki CZ, Arvaniti CC, Voumvourakis KI, Kostopanagiotou GG. Factors associated with the presence of postoperative headache in elective surgery patients: a prospective single center cohort study. J Anesth. 2017;31:225–36. doi: 10.1007/s00540-016-2285-z 27864621

