# Supplementary material for: Changes in blood catecholamines during induction of general anesthesia in patients with post-induction hypotension undergoing laparoscopic cholecystectomy: A single-center prospective cohort study
Source: PLoS One. 2024 Jun 25;19(6):e0305980. doi: 10.1371/journal.pone.0305980 (PMC11198742; doi:10.1371/journal.pone.0305980)
Supplement: S1 File — (PDF) [file pone.0305980.s001.pdf]

西南医科大学附属医院临床试验伦理委员会批文

受理号：KY2021293

项目名称	腹腔镜胆囊切除术患者全身麻醉诱导后低血压危险因素分析		
项目来源	自选课题		
研究单位	西南医科大学附属医院		
承担科室	麻醉科	主要研究者	王晓斌
审查类别	初审审查	审查方式	口全会 ☑快速 口紧急
审查日期	2021年12月16日	审查地点	NA

批准文件：

☑ 研究方案（版本号：1.0；版本日期：2021年09月07日）

☑ 知情同意书（版本号：1.0；版本日期：2021年09月08日）

审查意见：

根据ICH-GCP、中国GCP及相关法律、法规的规定，经本伦理委员会审查，同意按所批准的临床研究方案、知情同意书开展本项研究。

研究过程中注意事项（请仔细阅读）：

1.请遵循GCP原则，自觉接受国家有关法律和法规约束，遵循伦理委员会批准的方案开展临床研究，保护受试者的健康与权利；2.研究开始前，请申请人完成临床试验注册；3.所有资料未经伦理委员会批准，不得做任何修改；4．从批准之日起，应每年向本伦理委员会提交年度/定期跟踪审查报告，请在持续审查日期到期前一个月提出持续审查的申请；5．试验过程中发生以下情况应及时报告：①发生任何严重不良事件请立即（24小时内）报告；②违反研究方案；③暂停/终止研究；6．研究完成后提交总结报告；7.本批件自批准之日起一年内有效，逾期则自动废止。

审查结论：同意

有效期	1年	跟踪审查频率	12个月

地址：泸州市太平街25号　邮编：646000　联系人：张增瑞/何坤　电话：0830-3165273/973

伦理委员会主任委员（签章）：陈正君　西南医科大学附属医院临床试验伦理委员会（盖章）

2021年12月16日
