# Supplementary material for: Changes in blood catecholamines during induction of general anesthesia in patients with post-induction hypotension undergoing laparoscopic cholecystectomy: A single-center prospective cohort study
Source: PLoS One. 2024 Jun 25;19(6):e0305980. doi: 10.1371/journal.pone.0305980 (PMC11198742; doi:10.1371/journal.pone.0305980)
Supplement: S2 File — (PDF) [file pone.0305980.s002.pdf]

Project Name: Risk prediction of post-induction hypotension and analysis of blood catecholamine concentration during induction in patients undergoing laparoscopic cholecystectomy under general anesthesia

Project Leader：Xiaobin Wang

Project executor：Affiliated Hospital of Southwest Medical University

Dear participants:

You are invited to participate in the study on risk prediction of post-induction hypotension and analysis of blood catecholamine concentration during induction in patients undergoing laparoscopic cholecystectomy under general anesthesia, which is supported by the Affiliated Hospital of Southwest Medical University. Please read this informed consent carefully and make a careful decision whether to participate in this study. It is entirely your choice to participate in this study. As a subject, you must give your written consent before joining the clinical study. When your research doctor or researcher discusses the informed consent form with you, you can ask him/her to explain what you don't understand. We encourage you to have a full discussion with your family and friends before making a decision to participate in this study. You have the right to refuse to participate in the study or withdraw from the study at any time without being punished or losing your due rights. If you are participating in other studies, please inform your research doctor or researcher. The background, purpose, research process and other important information of this study are as follows:

1. The research background

Cholecystolithiasis is a common benign disease of the gallbladder, and its incidence has shown a younger trend. Surgery is the main method to treat cholecystolithiasis. Laparoscopic cholecystectomy is widely used in clinic because of its advantages of small trauma and rapid recovery. At present, laparoscopic cholecystectomy mostly uses general anesthesia. PIH (post-induction hypotension), as one of the complications of general anesthesia, can cause postoperative acute renal

function damage, myocardial injury, ischemic stroke and even death of patients. Some studies have shown that PIH is also associated with longer hospital stay, postoperative surgery related incidence rate, and even mortality in patients undergoing general, neurological, or cardiovascular surgery. In this study, we aim to analyze the PIH-related risk factors of patients undergoing laparoscopic cholecystectomy in our hospital prospectively, to provide reference for the prevention and treatment of PIH, and to provide a more optimized and individualized treatment plan to reduce the complications after laparoscopic cholecystectomy.

2. Research objectives

2.1. To explore the risk factors of hypotension induced by general anesthesia in patients undergoing laparoscopic cholecystectomy and establish relevant predictive models;

2.2 To explore the correlation between hypotension induced by general anesthesia and postoperative complications in patients undergoing laparoscopic cholecystectomy;

2.3 To explore the relationship between the change trend of catecholamine and hypotension during induction of laparoscopic cholecystectomy;

2.4 To provide a more optimized and individualized anesthesia scheme to reduce the occurrence of hypotension induced by laparoscopic cholecystectomy.

3. Research Protocol

3.1 How many people will participate in this study?

About 500 people will participate in the study, all at our hospital (Affiliated Hospital of Southwest Medical University).

3.2 Study procedure

3.2. 1 Preoperative evaluation indicators

When you agree to participate in this study, we will ask you some demographic data, including age, sex, body mass index (BMI), ASA grade, chronic disease, preoperative history of hypertension, creatinine, eGFR and basic blood pressure and other information. please answer truthfully. This information is for data analysis only and will not be disclosed.

Researcher signature;____

Date:______________________

3.2.2 Intraoperative evaluation indicators

We will record the incidence of hypotension after induction, as well as the dose of propofol, sufentanil and cisatracurium in each patient during anesthesia induction, record the heart rate, systolic pressure and diastolic pressure of the patient before anesthesia induction (T0), before tracheal intubation (T1), and 3-5 minutes after tracheal intubation (T2), and record the initial carbon dioxide pressure (PETCO2) after intubation. And ensure the stability of vital signs during the operation.

3.2.3 Postoperative evaluation indicators

Postoperative related indicators include nausea and vomiting, shoulder and back pain, headache and urinary retention, time of first exhaust and defecation, time of drainage tube removal, time of postoperative discharge, etc. These data will be extracted and analyzed by asking the patients, family members, tube bed doctors or consulting the electronic anesthesia record system. The postoperative sedation level was evaluated by Ramsay score. The specific scoring criteria are: 1=the patient shows anxiety and anxiety; 2=The patient is sober, quiet and cooperative; 3=The patient is lethargic, but responds quickly to the command; 4=The patient is in a light sleep state and can wake up quickly; 5=the patient is asleep and insensitive to loud calls; 6=The patient is in deep sleep and has no response to stimulation. AIS sleep scale was used to evaluate the patients' postoperative sleep status 3 days after surgery. The scale consists of 7 items, each of which is divided into four levels: 3=serious; 2=significant; 1=slight; 0 - No problem/normal, the total score is the sum of all items. The degree of insomnia was analyzed according to the total score of AIS scale: when the total score of the patient was 0 to 3, it was considered that there was no sleep disorder; When the total score of the patient is 4-5, they think they may have sleep problems and need guidance and help; When the total score of the patient is 6 or above, it is considered that the patient has insomnia and needs professional treatment. In our study, once the patient scores $\geq$ 4 points, it is determined to be positive, including suspected and symptomatic patients. This study only included the most severe insomnia of patients within 3 days into the analysis. This information is only for data analysis and will not be disclosed.

3.3 How long will this study last?

Researcher signature;____

Date:_______________________

This study is planned to be carried out from February 2022 to November 2022. All test scores were completed one day before operation and three days after operation. You can choose to drop out of the study at any time without losing any of the benefits you should have earned. However, if you decide to withdraw from this study, you are encouraged to consult your doctor first. If you have a serious adverse event, or if your study physician feels that continued participation is detrimental to your interests, he or she may decide to let you out of the study.Sponsors or regulators may also terminate the study during the study period. However, your withdrawal will not affect your normal medical treatment and rights and interests.

3.4 Information and biological samples collected in the study

In this study, blood samples can be collected according to the patient's physical indicators. If you agree to participate in this study, we will number each subject and create a medical record file. During the research, we may need to collect some blood samples from you. Before and after anesthesia induction, the patient will draw 3ml of blood into the anticoagulant tube for 3 times. Your sample is only used for clinical research.

4. Risk and benefit

4.1 What are the risks of participating in this study?

During the study, your sample collection process may have some very small risks, including transient pain and needle sickness. All your information will be kept confidential. We will try our best to protect the information you provide from being disclosed. However, we cannot guarantee the absolute security of information. At the same time, you can rest at any time during the study. You can withdraw from the study at any time during the study. If you have any discomfort, new changes in your condition, or any unexpected situation during the study, whether it is related to the study or not, you should notify your doctor in time, and the doctor will make a judgment and give appropriate treatment.

4 .2 What are the benefits of participating in the research?

Direct benefit: establish relevant risk factors of PIH, and conduct early intervention through model prediction to reduce the incidence of PIH. Potential benefits: testing your sample will help to observe the fluctuation of catecholamine

Researcher signature;____________________________________

Date:____________________

during surgery, provide necessary suggestions for your treatment, or provide useful information for disease research. We hope that the information you get from participating in this study will benefit more patients in the future.

5. Alternative treatment options

In addition to participating in this study, you can also receive routine treatment from doctors, such as maintain hemodynamic stability, etc.

6. The use of research results and the confidentiality of personal information

With the understanding and assistance of you and other subjects, the results of the research through this project may be published in medical journals, but we will keep your research records confidential according to legal requirements. The personal information of the research subjects will be strictly confidential, and your personal information will not be disclosed unless required by relevant laws. If necessary, the government management department, the hospital ethics committee and other relevant researchers can access your data according to regulations.

7. On research expenses and related compensation

7 . 1 Cost of drugs / instruments for research and related examinations

The drugs involved in this trial are routinely charged for anesthesiology, do not involve additional drugs and instruments, and will not incur additional costs in the course of normal treatment.

7 .2 Compensation for participation in the study

There is no additional compensation for participating in this study

7.3 Compensation / compensation after injury

The drugs and related scales in this study usually have no injuries and sequelae. In case of injury related to this study, you can accept the free treatment provided by the Affiliated Hospital of Southwest Medical University, or make compensation/ compensation according to the relevant laws of China.

8.Participant 's rights and related matters needing attention

8.1 Participant's rights

Researcher signature;____

Date:_______________________

You volunteered to take part in the study, if you decide not to participate in this study, it will not affect the other treatments you should receive. If you decide to participate, you will be required to sign this written informed consent form. You have the right to withdraw from the trial at any stage without discrimination or unfair treatment, and your corresponding medical treatment and rights and interests will not be affected.

8.2 Precautions

As a subject, you need to provide true information about your medical history and current physical condition; Inform the research doctor of any discomfort during this study; Do not take restricted drugs, food, etc; Tell the research doctor whether he has participated in other studies recently or is currently participating in other studies.

9. Relevant access for information

If there is any important new information during the study that may affect your willingness to continue to participate in the study, your doctor will notify you in time. If you want to know about your own research data or the findings of this study after the end of the study. You can ask any questions about this study at any time and get corresponding answers. Please contact the researchers at 19162632531/18715848933.

The Ethics Committee has reviewed and approved this study. If you have any questions related to your rights/ interests, or if you want to reflect the difficulties, grievances and concerns encountered in participating in this study, or would like to provide comments and suggestions related to this study, please contact the Ethics Committee of the affiliated Hospital of Southwest Medical University at 0830-3165273/ 3165972, email: xnydfyirb@sina.com.

Researcher signature;____

Date:______________________

Participant Signature Page

Informed Consent Statement :

I have been informed of the purpose, background, process, risks and benefits of this research. I have enough time and opportunity to ask questions, and I am satisfied with the answers.

I was also told who I should contact when I have questions, want to reflect difficulties, concerns, suggestions for research, or want to obtain further information or provide assistance for research.

I have read this informed consent form and agreed to participate in this study.

I know that I can choose not to participate in this research, or withdraw from this research at any time during the research period without any reason.

I already know that if my condition gets worse, or if I have a serious adverse event, or if my research doctor feels that it is not in my best interest to continue to participate in the study, he or she will decide to let me out of the study.

The funders or regulators may terminate the study during the study without my consent. If this happens, the researcher will inform me and discuss my other options with me.

I will get a copy of this informed consent form, which contains the signatures of me and the researcher.

Participant signature;
Date:______________________

Independent witness signature;
Date:______________________

(Note: if the participant cannot read the informed consent form,an independent witness is required to prove that the researcher has informed the participant of all the contents of the informed consent form, and the independent witness is required to sign and sign the date)

Researcher signature;____

Date:_______________________
