# Supplementary material for: Changes in blood catecholamines during induction of general anesthesia in patients with post-induction hypotension undergoing laparoscopic cholecystectomy: A single-center prospective cohort study
Source: PLoS One. 2024 Jun 25;19(6):e0305980. doi: 10.1371/journal.pone.0305980 (PMC11198742; doi:10.1371/journal.pone.0305980)
Supplement: S3 File — (PDF) [file pone.0305980.s003.pdf]

Approval of Clinical trial Ethics Committee of affiliated Hospital of Southwest Medical University

	Risk factors of hypotension after induction of general anesthesia in patients undergoing laparoscopic cholecystectomy		
Project fund	Optional subject		
Research institute	Affiliated Hospital of Southwest Medical University		
Undertaking department	Department of Anesthesiology	Project Leader	Xiaobin Wang
Review category	Initial review	Mode of review	☼ Plenary session 🏳 fast 🏳 urgent
Review date	December 16, 2021	Review place	The office of GCP

Approved document :

Study protocol (Version number: 1 .0; Version date: 2020-09- 07)

Informed consent form (Version number: 1 .0; Version date: 2020-09- 08)

Review comments:

In accordance with the provisions of ICH-GCP, China GCP and relevant laws and regulations, and reviewed by the Ethics Committee, agreed to conduct this study in accordance with the approved clinical research plan and informed consent form.

Notes during the study (please read carefully): 1. Please follow the GCP principles, consciously accept the relevant national laws and regulations, follow the program approved by the Ethics Committee to conduct clinical research, and protect the health and rights of the participants. 2. Before the start of the study, please complete the registration of clinical trials. 3. All materials may not be modified without the approval of the Ethics Committee. 4. From the date of approval , an annual / periodic follow-up review report shall be submitted to the Ethics Committee annually. Please submit an application for continuous review one month before the expiration of the date of continuous review. 5. The following situations should be reported in time during the study:⑴ any serious adverse events should be reported immediately (within 24 hours) ; ⑵ violation of the research scheme; ⑶suspension / termination of the study. 6. Submit a summary report after the completion of the study. 7. This approval is valid for one year from the date of approval. If it expires, it will be annulled automatically.

Review conclusion: agree

Validity period :	one year	Follow- up review frequency:	12 months

Add: 25 Taiping street, Luzhou city Postal code: 646000

Contact name: zengrui Zhang Tel :0830-3165273

Chairman of the Ethics Committee (signature)： zhengjun Cheng

Clinical trial Ethics Committee of the affiliated Hospital of Southwest Medical University (sealed
