# Supplementary material for: Changes in blood catecholamines during induction of general anesthesia in patients with post-induction hypotension undergoing laparoscopic cholecystectomy: A single-center prospective cohort study
Source: PLoS One. 2024 Jun 25;19(6):e0305980. doi: 10.1371/journal.pone.0305980 (PMC11198742; doi:10.1371/journal.pone.0305980)
Supplement: S4 File — (PDF) [file pone.0305980.s004.pdf]

A translation of our trial protocol

Project Name: Risk factors of hypotension induced by general anesthesia in patients undergoing laparoscopic cholecystectomy

Research Institute: Affiliated Hospital of Southwest Medical University

Project Leader：Xiaobin Wang

Project executor：Affiliated Hospital of Southwest Medical University

1、Research background

Cholecystolithiasis is a common benign disease of the gallbladder, and its incidence has shown a younger trend. Surgery is the main method to treat cholecystolithiasis. Laparoscopic cholecystectomy is widely used in clinic because of its advantages of small trauma and rapid recovery. At present, laparoscopic cholecystectomy mostly uses general anesthesia. PIH (post-induction hypotension), as one of the complications of general anesthesia, can cause postoperative acute renal function damage, myocardial injury, ischemic stroke and even death of patients. Some studies have shown that PIH is also associated with longer hospital stay, postoperative surgery related incidence rate, and even mortality in patients undergoing general, neurological, or cardiovascular surgery. In this study, we aim to analyze the PIH-related risk factors of patients undergoing laparoscopic cholecystectomy in our hospital prospectively, to provide reference for the prevention and treatment of induced hypotension, and to provide a more optimized and individualized treatment plan to reduce the complications after laparoscopic cholecystectomy.

2、 Research purpose

2.1 To explore the risk factors of hypotension induced by general anesthesia in patients undergoing laparoscopic cholecystectomy and establish relevant predictive models;
2.2 To explore the correlation between hypotension induced by general anesthesia and postoperative complications in patients undergoing laparoscopic cholecystectomy;

2.3 To explore the relationship between the change trend of catecholamine during induction and hypotension after laparoscopic cholecystectomy;
2.4 To provide a more optimized and individualized anesthesia scheme to reduce the occurrence of hypotension induced by laparoscopic cholecystectomy.

3、 Research overview

3.1 Overall research design and plan

This study is a prospective study. It is planned to include 500 patients who are scheduled to undergo laparoscopic cholecystectomy. The diagnosis types include cholecystitis, gallbladder polyps and gallstones.
3.2.1 Inclusion criteria
(1) age > 18 years;
(2) American Society of Anesthesiologists (ASA) physical status I–III; and
(3) elective laparoscopic cholecystectomy was performed under general anesthesia.

3.2.2 Exclusion criteria

(1) refused to involve in this trial at any time;

(2) received a conversion to open surgery;
(3) had severe preoperative comorbidities, including New York Heart Association(NYHA) class III–IV, moderate to severe impairment of pulmonary ventilation, child C grade of liver function, and stage III renal insufficiency;
(4) had a history of peripheral arterial diseases or atherosclerosis;
(5) opioid abusers or addiction to alcohol and other drugs;
(6) had any cardiac rhythm other than sinus, and had cardiomyopathy;
(7) poor compliance and loss of follow-up.

3.3 Grouping method and research category
This trial is a prospective study, which is divided into hypotension group and non-hypotension group.

3.4 Research steps and relevant examination

In strict accordance with the inclusion and exclusion criteria, after signing the informed consent form, the anesthesiologist will measure the baseline blood pressure of the patient before the operation, and the non-invasive arterial blood pressure will be used to measure the baseline blood pressure of the patient before the induction. At the same time, the height, weight, BMI, ASA grade, basic disease, and other characteristics of each patient were understood through the clinical digital anesthesia record sheet. The radial artery blood was collected from all patients at three time points before anesthesia induction (T0), after anesthesia induction (T1), and after tracheal intubation (T2). The blood samples of 80 patients were randomly selected for examination, and the changes of blood catecholamine before and after induction were observed, combined with the characteristics of patients before and after operation and the anesthetic use during operation, so as to understand the relationship between the induced hypotension and the above indicators, and provide useful information for disease research.

3.5 Observation indicators

(1) The occurrence of hypotension after induction.

(2) Heart rate, systolic blood pressure and diastolic blood pressure (perioperative period)

(3) Changes of blood catecholamines in some patients before and after anesthesia induction

(4) Use of narcotic drugs.

(5) Patient age, sex, weight, BMI, ASA grade, etc.

(6) Operation duration and postoperative adverse reactions.

4、Adverse event observation

4.1 Possible adverse events

(1) When blood samples were taken, there were transient palpitations, cold sweats and other dizziness reactions;

(2) Postoperative anxiety due to pre-induction evaluation and blood sampling.

4.2 Risk prevention and treatment

(1) Pay close attention to the patient's vital signs during sampling, and seek the help of professional psychologist at any time to avoid stress reaction

(2) Ask the physical and mental health doctor to conduct psychological counseling, confide and vent to avoid aggravating anxiety.

5、Statistical analysis

5.1 Inclusion of sample content;

Patients who underwent simple laparoscopic cholecystectomy from February 2022 to November 2022 in the General Department of Hepatobiliary Surgery and Surgery of the Affiliated Hospital of Southwest Medical University were included.

5.2 Statistics and analysis of research data

Statistical analysis was performed using R4.0.5. Categorical variables are expressed as counts and percentages (%), while continuous variables are expressed as mean ± standard deviation or median and quartile ranges. The independent sample t-test was used to compare the parameter values of the two groups, the Mann–Whitney U-test was used to compare the non-parameter values of the two groups, and the chi-square test was used to compare the classified variables. Variables with a p-value ≤ 0.1 were included in the multivariate logistic regression model to identify independent risk factors and then the nomogram of the prediction model was constructed. All tests were two-sided, and the significance level was set at $P < 0.05$, the ROC curve and the area under the curve (AUC) was drawn and calculated to evaluate the discrimination. Then, the calibration degree of the prediction model was evaluated by Hosmer-Lemeshow test and the calibration curve, and finally the decision curve analysis (DCA) was used to evaluate the clinical net benefit.

6、 Research related ethics

6.1 Review by the Ethics Committee

The protocol, the written informed consent form and the data directly related to the subject were submitted to the ethics committee, and the study was officially carried out after obtaining the written approval of the ethics committee. Inform the Ethics Committee in writing when the study is suspended or completed; Report all changes in the research work (such as the revision of the protocol or informed consent number) to the Ethics Committee in a timely manner, and do not implement these changes without the approval of the Ethics Committee, unless they are made to

eliminate the obvious and direct risks to the subjects. In such cases, the Ethics Committee will be informed.

6.2 Informed consent

6.2.1 Procedures for obtaining informed consent

The researcher must provide the subject or his legal representative with an easy-to-understand informed consent form approved by the ethics committee, and give the subject or his legal representative sufficient time to consider the study. The subject shall not be included in the group until the signed written informed consent form is obtained from the subject. During the participants' participation, all updated informed consent forms and written information will be provided to the subjects. The informed consent form shall be kept as an important document of the clinical trial for future reference.

7、 Confidentiality measures

The results of the research through this project may be published in medical journals, but we will keep the patient's information confidential according to the requirements of the law. Unless required by relevant laws, the patient's personal information will not be disclosed. When necessary, the government management department, the hospital ethics committee and its relevant personnel can consult the patient's information according to the regulations.

8、 Expected progress and completion date of the research project

February 2022 - November 2022: complete the collection of formal experimental data;

November 2022 - January 2023: summarize data and write papers;

January 2023 - February 2023: paper revision, submission and publication.

9、 References

[1]. Saab, R., et al., Failure to detect ward hypoxaemia and hypotension: contributions of insufficient assessment frequency and patient arousal during nursing assessments.

British Journal of Anaesthesia, 2021. 127(5): p. 760-768.
[2]. Filiberto, A.C., et al., Intraoperative hypotension and complications after vascular surgery: A scoping review. Surgery, 2021. 170(1): p. 311-317.
[3]. Chen, L., et al., Observer's Assessment of Alertness/Sedation-based titration reduces propofol consumption and incidence of hypotension during general anesthesia induction: A randomized controlled trial. Science Progress, 2021. 104(4): p. 003685042110523.
[4]. Khan, A.I., et al., The impact of fluid optimisation before induction of anaesthesia on hypotension after induction. Anaesthesia, 2020. 75(5): p. 634-641.
[5]. Dai, S., et al., A retrospective cohort analysis for the risk factors of intraoperative hypotension. International Journal of Clinical Practice, 2020. 74(8).
[6]. Assen, S., B. Jemal and A. Tesfaye, Effectiveness of Leg Elevation to Prevent Spinal Anesthesia-Induced Hypotension during Cesarean Delivery in the Resource-Limited Area: Open Randomized Controlled Trial. Anesthesiology Research and Practice, 2020. 2020: p. 1-8.
[7]. Sharkey, A.M., et al., Comparison of Intermittent Intravenous Boluses of Phenylephrine and Norepinephrine to Prevent and Treat Spinal-Induced Hypotension in Cesarean Deliveries. Anesthesia & Analgesia, 2019. 129(5): p. 1312-1318.
